# Post Laparoscopy Pain Reduction Project I (POLYPREP I): intraperitoneal normal saline instillation—a randomised controlled trial

**DOI:** 10.1186/s12905-022-01696-z

**Published:** 2022-04-12

**Authors:** Aizura Syafinaz Ahmad Adlan, Jerilee Mariam Khong Azhary, Hairel Zulhamdi Mohd Tarmidzi, Maherah Kamarudin, Raymond Chung Siang Lim, Doris Sin Wen Ng

**Affiliations:** 1 Department of Obstetrics and Gynaecology, Faculty of Medicine, University of Malaya, 50603 Kuala Lumpur, Malaysia; 2 Department of Obstetrics and Gynaecology, University Malaya Medical Centre, Kuala Lumpur, Malaysia

**Keywords:** Laparoscopic surgery, Gynaecological surgery, Postoperative pain

## Abstract

**Objectives:**

To evaluate the effect of intraperitoneal normal saline instillation (INSI) of 15 mL/kg body weight on postoperative pain after a gynaecological laparoscopic procedure.

**Design:**

Randomised controlled trial.

**Setting:**

University Hospital in Kuala Lumpur, Malaysia.

**Participants:**

Patients aged 18–55 years, with American Society of Anaesthesiologists (ASA) classification I–II, scheduled for an elective gynaecological laparoscopic procedure for a benign cause.

**Intervention:**

The patients were randomly allocated to two groups. In the intervention group, 15 mL/kg body weight of normal saline was instilled intraperitoneally, while the control group received the conventional combination of open laparoscopic trocar valves with gentle abdominal pressure to remove the retained carbon dioxide.

**Main outcome measures:**

The outcomes measured were the mean pain scores for shoulder and upper abdominal pain at 24 h, 48 h, and 72 h postoperatively.

**Results:**

A total of 68 women completed the study, including 34 women in each group. There was no difference in the shoulder pain score at 24 h, 48 h, and 72 h postoperatively. However, a significant improvement in the upper abdominal pain score after 48 h (95% confidence interval [CI] 0.34–1.52, p = 0.019) and 72 h (95% CI 0.19–0.26, p = 0.007) postoperatively were observed.

**Conclusions:**

INSI of 15 mL/kg body weight does not lower postoperative shoulder pain compared to no fluid instillation. A modest pain score improvement was observed in the upper abdominal area at 48 h and 72 h after surgery. An INSI of up to 30 mL/kg body weight may be required to eliminate shoulder pain. Care must be taken before administering a higher amount of INSI, considering the potential risk of peritoneal adhesions.

*Clinical registration* ISRCTN Identifier: 87898051 (Date: 26 June 2019) https://doi.org/10.1186/ISRCTN87898051

## Background

Postoperative pain should be addressed effectively as it can complicate a patient’s recovery process. Inadequate postoperative pain control is associated with impaired quality of life, delayed recovery, and prolonged use of opioids, with a concurrent increase in healthcare costs [1]. Despite the emergence of many intraoperative techniques and manoeuvres to reduce postoperative pain, pain after gynaecological laparoscopic procedures remains high [2–4]. To date, at least 11 strategies have been demonstrated to be effective in controlling post-laparoscopy pain [5, 6]. One of these strategies involves instilling fluid into the peritoneal cavity [5–8].

Intraperitoneal normal saline instillation (INSI) is a pain reduction method that functions by washing out residual carbon dioxide [7–9]. Moreover, it can act as a physiological buffer whereby residual carbon dioxide is dissolved in the fluid [9–11]. The removal of the retained carbon dioxide is crucial because it can be converted to carbonic acid via the peritoneal carbonic anhydrase enzyme [12]; this process reduces the pH level, causing irritation or damage to the diaphragmatic peritoneal nerves, leading to pain [9–12].

In gynaecological surgery, this pain reduction technique has been implemented in several different studies. It was found that INSI significantly reduced shoulder and upper abdominal pain compared to no fluid instillation [7–11, 13]. Although these studies were performed by the same researchers, the amount of fluid instilled varied between 15–30 mL/kg body weight [10, 11]. Under these circumstances, a 60 kg weighted woman could either be receiving 900 mL (15 mL/kg) or up to 1800 mL (30 mg/kg) of INSI, which is twice the amount.

A laparoscopic surgeon should take extra precautions when considering INSI. The peritoneum is composed of a single layer of mesothelial cells and is responsible for facilitating the gliding of the bowel via its microvilli, glycosaminoglycans, and surfactants [14]. Peritoneal mesothelial cells react to trauma and chemical irritants quickly [15, 16]. Normal saline, for example, detaches mesothelial cells, leading to a loss of fibrinolytic activity [17]. This negative effect from saline has been confirmed in vitro [18–20] and in vivo [21] Additionally, studies have demonstrated that normal saline dwelling in the peritoneal cavity during peritoneal dialysis or laparotomy wash-out can lead to the formation of peritoneal adhesions [20, 21]; these adhesions can cause chronic and life-threatening symptoms such as abdominal distension, pain, nausea, and abnormal bowel movements [22]. In animal models, intraperitoneal normal saline increases lymphatic flow [23]. In this study, we attempted to produce the pain relief effect of INSI by fixing the lowest amount of normal saline to be instilled into the peritoneal cavity at 15 mL/kg body weight, which was the lowest previously tested dose [10, 11, 13].

## Methods

### Setting and participants

This study was carried out in the general operating theatre of the University Malaya Medical Centre, Kuala Lumpur, Malaysia. Written informed consent was obtained during patient visits to the gynaecology clinic a few weeks before the date of surgery or during ward admission a day before the surgery. Patients assigned to the American Society of Anaesthesiologists (ASA) physical classification [24] of I or II, aged between 18 and 60 years, and scheduled to undergo a laparoscopic procedure for benign gynaecological conditions were considered eligible for participation. The definitions of ASA are as follows: ASA I, normal healthy patient; ASA II, mild systemic disease without substantial functional limitations (body mass index < 40 kg/m^2^, well-controlled diabetes mellitus/mild hypertension, mild lung disease). Patients with known drug allergies to substances in parecoxib, celecoxib, paracetamol, or having received analgesia 12 h before the scheduled procedure were excluded. This study was registered and approved by the Ministry of Health Research and Ethics Committee (MREC) of the National Medical Research Register (NMRR) and the Clinical Research Centre, Malaysia (NMRR-19-1532-48232) on 06/03/2019 and the Medical Research Ethics Committee of the University Malaya Medical Centre (MREC ID No: 201926-7106) on 20/03/2019. Furthermore, the International Standardised Randomised Controlled Trial Number (ISRCTN) for this study was registered with an ID of ISRCTN87898051 (https://doi.org/10.1186/ISRCTN87898051), dated 26/06/2019. This trial was conducted in accordance with the Declaration of Helsinki (2000) for human studies.

### Randomisation and blinding

Women were assigned to two groups in a 1:1 ratio using a random-permuted block randomisation algorithm via a web-based system (www.randomization.com) by a research assistant (RA-A) who was not involved in the recruitment process. The same research assistance kept the master list for the randomised treatment allocation sequences. Concealment was performed using serially numbered opaque, sealed envelopes containing a paper with the legend ‘INSI’ or ‘Control’. The following available randomisation number was assigned to the patient once she consented to participate. Upon arrival at the theatre, the allotted envelope was subsequently given to the operating theatre nurse, who was not involved in managing the patient. The envelope was opened at the end of the surgery before the removal of laparoscopic trocars. Patients who withdrew from the study before surgery were replaced by the next consenting patient. However, patients who withdrew from the study at the end of surgery were counted as dropouts, and no replacement was made.

### Intervention

A standard preoperative anaesthesia form was completed in accordance with the Department of Anaesthesia’s protocol. All laparoscopic surgeries were performed in the general operation theatre at Universiti Malaya Medical Centre, Kuala Lumpur. No pre-medication or any form of analgesia was administered prior to the procedure. The standard anaesthetic agents used were propofol (1.5–2.5 mg/kg), fentanyl (1–2 mcg/kg), metoclopramide (10 mg), and atracurium (0.3–0.6 mg/kg). Anaesthesia was maintained with 50% nitric oxide, 50% oxygen, and 1–2% sevoflurane. No other analgesic was administered intraoperatively.

For each laparoscopy, while the patient was in the supine position, a sub-umbilical incision was made, and a 10-mm trocar was inserted sub-umbilically under direct vision. Pneumoperitoneum was created by insulating 2.5 L of carbon dioxide into the peritoneal cavity. Another 5-mm trocar was inserted into the right iliac fossa, left iliac fossa, or suprapubic region. At the end of the surgery, patients allocated to both groups were placed in the Trendelenburg position (30°). Subsequently, trial protocols were carried out as follows:

The intervention group (INSI): Intraperitoneal normal saline (15 mL/kg body weight) [10, 11] was infused at the upper part of the abdominal cavity evenly by the surgeon through a 5-mm trocar. The trocar sleeve valves were left open during the instillation of normal saline to allow carbon dioxide to escape from the abdominal cavity. The instilled normal saline solution was left in situ.

The control group: Trocar sleeve valves were left open to allow carbon dioxide to escape from the abdominal cavity with gentle abdominal pressure.

In both groups, the trocar incision site was closed in two layers using Vicryl 3/0 or Monosyn 3/0. Patients were then placed in a neutral position, and standard protocols of anaesthesia reversal were carried out.

### Postoperative management

The patients received standard postoperative care in the ward and were discharged according to the discretion of each managing team. A standard regime of analgesia was administered to all women, in which 1 g of paracetamol and 40 mg of parecoxib were administered intravenously, or 75 mg of suppository diclofenac acid was administered at the end of the surgery. This was followed by standard regular doses of 1 g paracetamol every 6 h for 5 days. Additional doses of analgesia (oral celecoxib 200 mg twice a day), when administered, were recorded.

### Measurement of pain score

The outcomes of this study were the severity of pain in the shoulder and upper abdominal areas at 24 h, 48 h, and 72 h after the laparoscopic surgery. It was measured using a 0–10 visual analogue score (VAS), where 0 = no pain and 10 = worst possible pain. The VAS has been adapted by the Ministry of Health Malaysia as a pain assessment tool [25].

Women rated their postoperative pain using the VAS at rest and during movement, at a specific location (shoulder and upper abdomen) and time (24 h, 48 h, and 72 h) postoperatively. The occurrence of nausea, vomiting, and abdominal distension was documented as secondary outcomes. The data were recorded by another research assistant (RA-B), who was blinded to the patients' assigned group. If the patients were discharged before day 3 (72 h), the patients were contacted over the phone by the research assistant RA-B, who was blinded to the assigned group of patients to obtain their pain scores.

### Statistical analysis

The sample size was calculated using the G*Power software version 3.1.94. Based on a previous study [11], the mean shoulder pain (standard deviation [SD]) of VAS at 48 h post laparoscopic procedure was pain 1.26 [1.95] and 3.21 [2.78] for INSI and control groups, respectively. With a two-sided significance level of 5% and power of 85%, the minimum number needed for each group was 28. By estimating a 20% dropout rate and rounding up, we planned to recruit 68 patients (34 women in each arm).

Statistical analysis was performed using standard parametric and non-parametric statistics with JMP Pro 14.1 (SAS Institute Inc., Cary, NC, USA). Data were expressed as mean ± SD or number (%). Fisher’s exact test was used to analyse categorical variables, while the independent sample t-test or one-way ANOVA test was used to analyse continuous variables. A two-sided p-value < 0.05, was considered as the threshold for significance.

## Results

### Patients’ characteristics

**Fig. 1:**
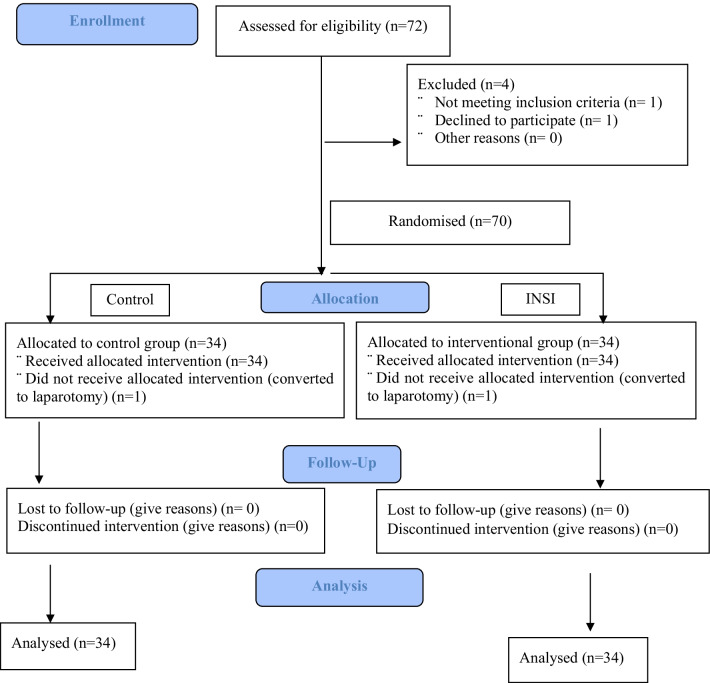
Consort flow chart

A total of 72 patients with benign gynaecological conditions scheduled for laparoscopic surgeries from April 2019 to October 2019 were recruited. One patient declined participation, and one patient was excluded based on the exclusion criteria (allergy to parecoxib, n = 1). Subsequently, 70 patients were included; however, two patients had their laparoscopy procedure converted to laparotomy (control, n = 1; INSI, n = 1). Therefore, 68 patients completed the study (Fig. 1). Baseline characteristics, including age, parity, marital status, ethnicity, body mass index, ASA classification, and indication for surgery, were comparable between the groups. The surgical outcomes, including operative time, drain insertion, estimated blood loss, number of trocars inserted, and length of postoperative hospital stay were not significantly different between the groups (Table 1).

**Table 1:** Subject characteristics and surgical outcomes

Variables	Control	Intraperitoneal normal saline (INSI)
	n = 34	n = 34
Age (mean ± SD)	36.1 (± 8.9)	35.6 (± 8.9)
Parity, n (%)		
Nulliparous	16 (47.1%)	12 (35.3%)
Parous	18 (52.9%)	22 (64.7%)
Ethnicity n (%)		
Malay	19 (55.9%)	22 (64.7%)
Chinese	8 (23.5%)	3 (8.8%)
Indian	4 (11.8%)	7 (20.6%)
Others	3 (8.8%)	2 (5.9%)
Body mass index (kg/m^2^) (mean ± SD)	25.2 (± 5.4)	27.9 (± 6.7)
ASA classification, n (%)		
I	22 (64.7%)	20 (58.8%)
II	12 (35.3%)	14 (41.2%)
Previous abdominal surgery, n (%)	10 (29.4%)	10 (29.4%)
Indication for surgery, n (%)		
Hysterectomy	0 (0%)	1 (2.9%)
Myomectomy	2 (5.9%)	1 (2.9%)
Ovarian cystectomy	8 (23.5%)	10 (29.4%)
Tubal ligation	2 (5.9%)	1 (2.9%)
Diagnostic laparoscopy	5 (14.7%)	2 (5.9%)
Salpingectomy	8 (23.5%)	13 (38.2%)
Salpingo-oophorectomy	5 (14.7%)	1 (2.9%)
TLHBSO	4 (11.8%)	4 (11.8%)
Operative time (min), (mean ± SD)	80.2 (± 37.2)	93.2 (± 40.5)
Estimated blood loss (mL), (mean ± SD)	409 (± 453)	365 (± 232)
Trocars inserted, n (%)		
2	4 (11.8%)	0 (0.0%)
3	25 (73.5%)	29 (85.3%)
4	5 (14.7%)	4 (14.7%)
Postoperative hospital stays (days), n (%)	1.8 (± 0.7)	1.8 (± 0.8)

ASA, American Society of Anaesthesiologists; INSI, intraperitoneal normal saline; SD, standard deviation; TLHBSO, total laparoscopic hysterectomy with bilateral salpingo-oophorectomy

### Shoulder pain

**Fig. 2:**
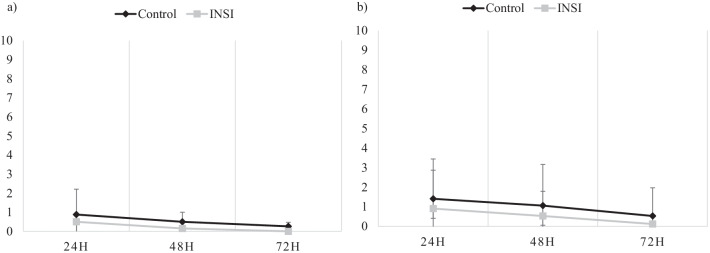
Mean visual analogue scores for shoulder pain at 24 h, 48 h, and 72 h after the procedure at **a** rest and **b** on movement. Data are presented as mean (standard deviation). The test of significance was performed by one-way ANOVA test. H, hours; *p < 0.05; INSI, intraperitoneal normal saline instillation

The severity of shoulder pain at 24 h, 48 h, and 72 h post-laparoscopic surgery was not significantly different between the two groups, both at rest and during movement, as shown in Table 2. However, the mean pain score recorded in the control group was persistently higher throughout the study (Fig. 2).

**Table 2:** Postoperative shoulder pain

Postoperative duration (h)	Control, n = 34		INSI, n = 34		p-value
	Pain score (mean ± SD)	95% CI	Pain score (mean ± SD)	95% CI	
At rest					
24	0.88 ± 1.49	0.36, 1.40	0.50 ± 1.33	0.04, 0.96	0.269
48	0.50 ± 1.14	0.10, 0.90	0.15 ± 0.50	− 0.03, 0.32	0.102
72	0.26 ± 0.90	− 0.05, 0.58	0.00 ± 0.00	0.00, 0.00	0.90
On movement					
24	1.41 ± 2.03	0.70, 2.12	0.91 ± 1.96	0.23, 1.60	0.305
48	1.06 ± 2.10	0.33, 1.79	0.53 ± 1.26	0.09, 0.97	0.212
72	0.53 ± 1.44	0.03, 1.03	0.12 ± 0.537	− 0.07, 0.31	0.123

Data expressed as mean ± SD (standard deviation) and 95% CI (confidence interval for mean). The test of significance was performed by one-way ANOVA test

CI, confidence interval; INSI, intraperitoneal normal saline; SD, standard deviation

### Upper abdominal pain

**Fig. 3:**
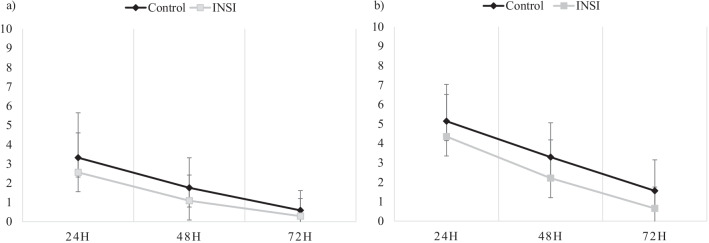
Mean visual analogue scores for abdominal pain at 24 h, 48 h, and 72 h after the procedure at **a** rest and **b** on movement. Data are presented as mean (standard deviation). The test of significance was performed by one-way ANOVA test. H, hours; *p < 0.05; INSI, intraperitoneal normal saline instillation

The severity of the upper abdominal pain after laparoscopic surgery was statistically insignificant at 24 h postoperatively. However, the pain score was significantly lower in the INSI group at 48 h (p = 0.019) and 72 h (p = 0.007) postoperatively during movement than those in the control group. The mean (± SD) pain score recorded at postoperative 48 h was 3.29 ± 1.77 (95% confidence interval [CI]: 0.30–2.68) and 2.21 ± 1.97 (95% CI: 0.34–1.52) in the control and INSI groups, respectively. After postoperative 72 h, the mean (± SD) pain score was 1.56 ± 1.5 (95% CI: 0.27–1.01) and 0.65 ± 1.1 (95% CI: 0.19–0.26) in the control and INSI groups, respectively (Table 3 and Fig. 3).

**Table 3:** Postoperative abdominal pain

Postoperative duration (h)	Control, n = 34		INSI, n = 34		p-value
	Pain score (mean ± SD)	95% CI	Pain score (mean ± SD)	95% CI	
At rest					
24	3.32 ± 2.33	0.40, 2.51	2.56 ± 2.05	0.35, 11.84	0.155
48	1.76 ± 1.56	0.27, 1.22	1.09 ± 1.33	0.23, 0.62	0.059
72	0.59 ± 1.02	0.18, 0.23	0.29 ± 0.91	− 0.02, 0.61	0.213
On movement					
24	5.14 ± 1.90	0.33, 4.51	4.35 ± 2.17	0.37, 3.56	0.101
48	3.29 ± 1.77	0.30, 2.68	2.21 ± 1.97	0.34, 1.52	0.019
72	1.56 ± 1.59	0.27, 1.01	0.65 ± 1.10	0.19, 0.26	0.007

Data expressed as mean ± SD (standard deviation) and 95% CI (confidence interval for mean). The test of significance was performed by one-way ANOVA test

CI, confidence interval; INSI, intraperitoneal normal saline; SD, standard deviation

### Nausea, vomiting, and analgesia requirement

There were no statistically significant differences in the incidence of nausea, vomiting, abdominal distension, and the need for additional analgesia. Specifically, no patient required additional analgesia in either group throughout the study duration. At postoperative 24 h, 7 patients (control, n = 3; INSI, n = 4) complained of nausea, and 3 (control, n = 2; INSI, n = 1) complained of vomiting. In terms of initiation of oral feed, 31 patients from the control group and 33 patients from the INSI group began to ingest meals per orally 24 h after surgery. At postoperative 24 h, 13 patients from the control group and 8 patients from the INSI group complained of abdominal distension, of which only 3 patients experienced distension up to 48 h after surgery (control, n = 2; INSI, n = 1). Majority of the patients from both the groups were able to pass flatus at postoperative 24 h (control, n = 28; INSI, n = 32). No other complications, such as fluid leakage through the incision site or infection, were observed in the interventional or control groups. Most patients were discharged between day 1 and day 2 post-surgery (control, n = 31; INSI, n = 27). Only one patient was discharged 4 days after surgery because of personal logistic issues.

## Discussion

### Main findings

In contrast to other studies, the results obtained from this trial demonstrated that 15 mL/kg of INSI did not significantly reduce shoulder pain compared to no fluid instillation at 24 h, 48 h, or 72 h post gynaecological laparoscopy procedure. However, similar to previous trials, our data revealed that upper abdominal pain was significantly improved at 48 h and 72 h after INSI of 15 mL/kg.

### Strengths and limitations

In our opinion, this trial was designed with adequate precaution to eliminate preventable bias and modestly fill the research gap on the efficacy of INSI 15 mL/kg body weight in reducing postoperative pain, the lowest dose tested by previous researchers.

Our study has certain limitations. First, the sample size of this study was relatively small, limiting the generalisation of the results. Second, the differences in patient characteristics were a source of bias that could affect the stability of the results. Moreover, although all surgeons were advised to actively evacuate the retained carbon dioxide with gentle pressure on the abdomen, some surgeons may have performed it better than others, contributing to another source of bias.

Another major limitation is lack of blinding of the laparoscopic surgeons. Complete blinding is not practical as the act of not instilling and instilling fluid is not concealable from the surgeons performing the surgery.

### Interpretation

Our findings were inconsistent with the findings of previous researchers, which could be due to the amount of INSI we chose to instil and/or characteristic differences in our patients.

We opted for a lower amount of INSI in order to minimise excessive indwelling normal saline in the peritoneal cavity. In the trials carried out by Tsai et al. [10, 11], the amount of saline instilled was up to 30 mL/kg body weight. The team suggested that intraperitoneal absorption is estimated to be approximately 30–60 mL/h [19, 26]; therefore, the concern about fluid shifts is reduced. However, the absorption rate cited was for Ringer’s Lactate [19, 26], whose composition differs from that of normal saline. The rate of intraperitoneal absorption of normal saline is still being investigated. However, when 1 L of normal saline was instilled into the peritoneal cavity laparoscopically, it was fully absorbed after 16 h [20, 27]. In our trial, the normal saline fluid instilled ranged between 500 and 1500 mL depending on the patient’s weight.

Excessive indwelling intraperitoneal normal saline promotes formation of peritoneal adhesions [20, 21, 28]. Despite its non-physiological composition [20] normal saline is commonly used to wash the abdominal cavity during open and laparoscopic surgeries to remove remnants of blood and other fluids [29]. However, complete removal of the fluid during surgery is not always possible. A previous study demonstrated that patients undergoing laparoscopic surgery had at least 25% of residual normal saline in the abdominal cavity despite the surgeon's best effort to evacuate it, and this residual saline undergoes gradual absorption [21]. However, it was observed that as the saline dwelling in the peritoneal cavity undergoes compositional changes, it becomes less biocompatible to mesothelial cells, promoting peritoneal adhesion. Additionally, elastase activity is also increased in the indwelling fluid, reflecting an intraperitoneal inflammatory reaction [28]. However, the use of normal saline continues, especially for postoperative pain reduction during laparoscopy procedures due to a vast amount of available data validating its safety and efficacy [7, 8, 10, 11, 13, 30, 31]. Furthermore, when Hartmann’s or Ringer’s lactate solution was used instead of normal saline, there was no noticeable improvement in pain score [32].

Second, compared to Tsai et al. [10], the patients’ characteristics in this trial showed a higher prevalence of nulliparous women (control, 47.1%; INSI, 35.3%) than multiparous women. Abdominal wall laxity significantly influences the type of pain perceived by the patient [33]. The shoulder and upper abdominal pain are results of distension and irritation of the peritoneum. Multiparous women tend to have less stretching and irritation of the peritoneum than nulliparous women. Therefore, it is reasonable to say that the rigid abdominal wall of nulliparous women leads to higher incidences of postoperative pain [33]. Additionally, other studies [34, 35] also reported being unsuccessful in reducing postoperative pain with some manoeuvres.

Other advantages of INSI include its potential to provide long-lasting and persistent pain relief [10]. Similar to previous trials, our data revealed that upper abdominal pain was significantly improved at 48 h and 72 h post-INSI of 15 mL/kg body weight. The rationale for observing pain scores up to 72 h was that pain after a laparoscopic procedure may last up to 3 days and even up to 5 weeks [32, 36, 37]. However, no studies have assessed pain scores beyond 48 h post gynaecological laparoscopic procedures. Here, we provide data on pain scores 72 h after gynaecological surgery.

## Conclusions

In conclusion, instillation of normal saline of 15 mL/kg body weight at the end of any benign gynaecological laparoscopic surgery does not improve shoulder pain. However, a modest improvement in pain score was observed in the upper abdominal area 48 h and 72 h post-procedure. The option to increase the amount of saline instillation to up to 30 mL/kg body weight in order to improve pain score should be carefully evaluated, considering that INSI may result in development of peritoneal adhesions even at lower amounts. Thus, a longitudinal controlled randomised trial should be conducted to address the occurrence of postoperative peritoneal adhesions after INSI.

## Data Availability

The datasets used and analysed during the current study are available from the corresponding author on reasonable request.

## Abbreviations

ASA: American Society of Anaesthesiologists
INSI: Intraperitoneal normal saline instillation
ISRCTN: International Standardised Randomised Controlled Trial Number
MREC: Ministry of Health Research and Ethics Committee
NMRR: National Medical Research Register
RA-A: First research assistant
RA-B: Second research assistant
VAS: Visual analogue score
